# Impaired functional exercise capacity and greater cardiovascular response to the 6-min walk test in congenital generalized lipodystrophy

**DOI:** 10.1186/s12872-022-02828-x

**Published:** 2022-08-25

**Authors:** Jorge Luiz Dantas de Medeiros, Bruno Carneiro Bezerra, Helen Rainara Araújo Cruz, Katarina Azevedo de Medeiros, Maria Eduarda Cardoso de Melo, Aquiles Sales Craveiro Sarmento, Marcela Abbott Galvão Ururahy, Lucymara Fassarella Agnez Lima, Alcebíades José dos Santos Neto, Josivan Gomes Lima, Vanessa Resqueti, Lucien Peroni Gualdi, Guilherme Fregonezi, Julliane Tamara Araújo de Melo Campos

**Affiliations:** 1 PneumoCardioVascular Lab/HUOL, Hospital Universitário Onofre Lopes, Empresa Brasileira de Serviços Hospitalares and Departamento de Fisioterapia, Universidade Federal do Rio Grande do Norte, Natal, Brazil; 2 Faculdade de Ciências da Saúde do Trairi, Universidade Federal do Rio Grande do Norte, Santa Cruz, RN Brazil; 3 Laboratório de Biologia Molecular e Genômica, Departamento de Biologia Celular e Genética, Centro de Biociências, Universidade Federal do Rio Grande do Norte, Natal, RN Brazil; 4 Departamento de Análises Clínicas e Toxicológicas, Faculdade de Farmácia, Universidade Federal do Rio Grande do Norte, Natal, RN Brazil; 5 Departamento de Medicina Clínica, Hospital Universitário Onofre Lopes (HUOL)/UFRN, Natal, RN Brazil; 6 Laboratório de Inovação Tecnológica em Reabilitação, Departamento de Fisioterapia, Universidade Federal do Rio Grande do Norte, Natal, Brazil

**Keywords:** Lipodystrophy, Metabolism, Cardiovascular response, Ankle-brachial index, Six-min walk test

## Abstract

**Background:**

Congenital Generalized Lipodystrophy (CGL) is an ultra-rare disease characterized by metabolic disorders. However, the evaluation of functional exercise capacity, cardiovascular (CV) response to exercise, and peripheral arterial disease (PAD) in CGL is scarce. Here we evaluated the performance and CV response to exercise and their association with PAD in CGL compared to healthy individuals.

**Methods:**

Twelve CGL and 12 healthy subjects matched for age and gender were included. Functional exercise capacity, CV response, and PAD were measured using the six-minute walk test (6MWT) and ankle-brachial index (ABI), respectively.

**Results:**

At baseline, CGL subjects showed reduced predicted walked distance (6MWD) (*p* = 0.009) and increased heart rate (HR), systolic (SBP), and diastolic (DBP) pressures compared to healthy subjects (*p* < 0.05). Most CGL subjects presented normal ABI values (1.0 ≤ ABI ≤ 1.4). Only 25% (n = 3) had ABI ≤ 0.9. CGL subjects did not present changes in ABI and blood pressure 12 months after metreleptin (MLP) replacement, but they walked a greater 6MWD than baseline (*p* = 0.04). Further, 6MWD and right ABI measurements were positively correlated in CGL subjects (*p* = 0.03). Right ABI negatively correlated with glucose, triglycerides, and VLDL-c (*p* < 0.05).

**Conclusions:**

We observed that CGL subjects had lower functional exercise capacity and higher cardiovascular effort for similar performance of 6MWT, suggesting that strategies for decreasing exercise effort in this population should be essential. Furthermore, better physical performance was associated with high ABI in CGL. Additional studies are needed to clarify leptin's role in preserving functional exercise capacity in CGL.

**Supplementary Information:**

The online version contains supplementary material available at 10.1186/s12872-022-02828-x.

## Introduction

Berardinelli-Seip Congenital Generalized Lipodystrophy (CGL) is a rare genetic syndrome categorized by the almost total decrease of fatty tissue since birth [1, 2]. CGL patients present increased serum levels of triglycerides and reduced levels of high-density lipoprotein (HDL), as well as leptin and adiponectin [3]. Further, they have insulin resistance, diabetes *mellitus* (DM), hepatosplenomegaly, and hepatic steatosis. At the morphological level, they present prominent musculature, prognathism, umbilical protrusion, acanthosis nigricans, and phlebomegaly [4–8]. Cardiovascular (hypertrophic cardiomyopathy, arterial hypertension, cardiovascular autonomic neuropathy, and atherosclerosis) and respiratory disturbances (respiratory muscle weakness) have been currently described in patients with CGL [4, 9–13]. Lipodystrophic subjects may have muscle dysfunction and several pathophysiological mechanisms may be related, including increased endoplasmic reticulum stress [14].

The *Rio Grande do Norte* (*RN*) state in Northeastern Brazil presents a high prevalence of CGL [15], and clinical, laboratory, and genetic data, as well as the causes of death, were previously described for this population [3, 8]. Although liver disease and infections were the first cause of mortality, Lima et al. (2018) found that cardiomyopathy and sudden cardiac arrest are important death causes. These comorbidities result in the very early death of CGL subjects [1]. Further, the same group found two CGL patients with bilateral occlusion of the femoral artery, and one patient died due to necropsy-confirmed myocardial infarction [8].

Considering that the clinical aspects of CGL may impair the respiratory, skeletal muscle, and cardiovascular systems [6, 9, 16, 17], functional exercise capacity would also be compromised and might be a significant clinical and prognostic clinical marker. The objective of this study was to assess the performance and cardiovascular response (CV) to the 6-min walk test (6MWT) in CGL individuals compared to controls. Moreover, we evaluated the occurrence of peripheral arterial disease (PAD) and its association with functional exercise capacity in CGL.

## Methods

### Study population and data collection

This longitudinal study was conducted from November 2018 to December 2019 in the *Seridó* region of Rio Grande do Norte Brazilian State. The sample size was not calculated since CGL is an ultra-rare disease with a prevalence of 1:1,000,000 inhabitants worldwide[18]. RN Brazilian state presents a well-known high prevalence of this metabolic disease [15].

Inclusion criteria were to be genetically or clinically diagnosed with CGL, older than 18 years of age, and able to understand the tests. CGL subjects of both sexes were recruited during the annual meeting promoted by *Associação de Pais e Pessoas com a Síndrome de Berardinelli do Estado do Rio Grande do Norte (ASPOSBERN*). ASPOSBERN is a non-profit association that contributes to managing CGL subjects diagnosed by qualified physicians and researchers from the Brazilian Research Group for Studies about the Genetics and Morphophysiological Features of Berardinelli-Seip Lipodystrophy [15]. ASPOSBERN annual meeting, in association with the actions of our research group, has a program of fraternization, guidance, and dissemination of information for parents and patients with CGL. During the event, clinically and/or genetically diagnosed CGL individuals were invited to participate in the survey by the health professionals of this research. The assessments are carried out in the attached places by the group of researchers.

Healthy individuals matched for age and gender who signed the WICF were recruited in the first 3 months of 2019 at the *Clínica Escola de Fisioterapia*, from *Faculdade de Ciências da Saúde do Trairi*, a campus of *Universidade Federal do Rio Grande do Norte* (*UFRN*). We excluded subjects who had previously had vascular diseases from both groups, such as unstable angina, myocardial infarction, chronic heart failure, uncontrolled systemic arterial hypertension (cutoff value of 140 × 90 mmHg), and psychiatric disorders or inability to perform the test. In addition, no CGL and diabetes diagnosis were also exclusion criteria for the control group.

The clinical, metabolic, and genetic features of CGL subjects were previously described by Lima et al. [8], Medeiros et al. [15], and Craveiro Sarmento et al. [3]. Some CGL subjects from our study started in 2016 using metreleptin (MLP) replacement to treat leptin deficiency complications, such as insulin resistance, DM, and hypertriglyceridemia during the survey. MLP replacement therapy was performed according to Musso et al. [19].

### Biochemical measurements in plasma

Blood samples were taken after eight hours of fasting. Serum was separated, stored at -80 °C, and analyzed at a later time. Triglycerides, glucose, and total cholesterol measurements in plasma were performed by Trinder’s method, according to the instructions from the Labtest protocols (Lagoa Santa, Brazil) and LABMAX PLENNO equipment (LABTEST, Lagoa Santa, Brazil) [20]. HDL-c was measured by selective precipitation of cholesterol loaded into very-low-density lipoprotein (VLDL-c) and LDL-c (19). LDL-c and non-HDL-c were calculated according to the Martin method [21]. These analyses were performed at Departamento de Análises Clínicas e Toxicológicas from UFRN.

### Six-min walk test (6MWT)

**Fig. 1:**
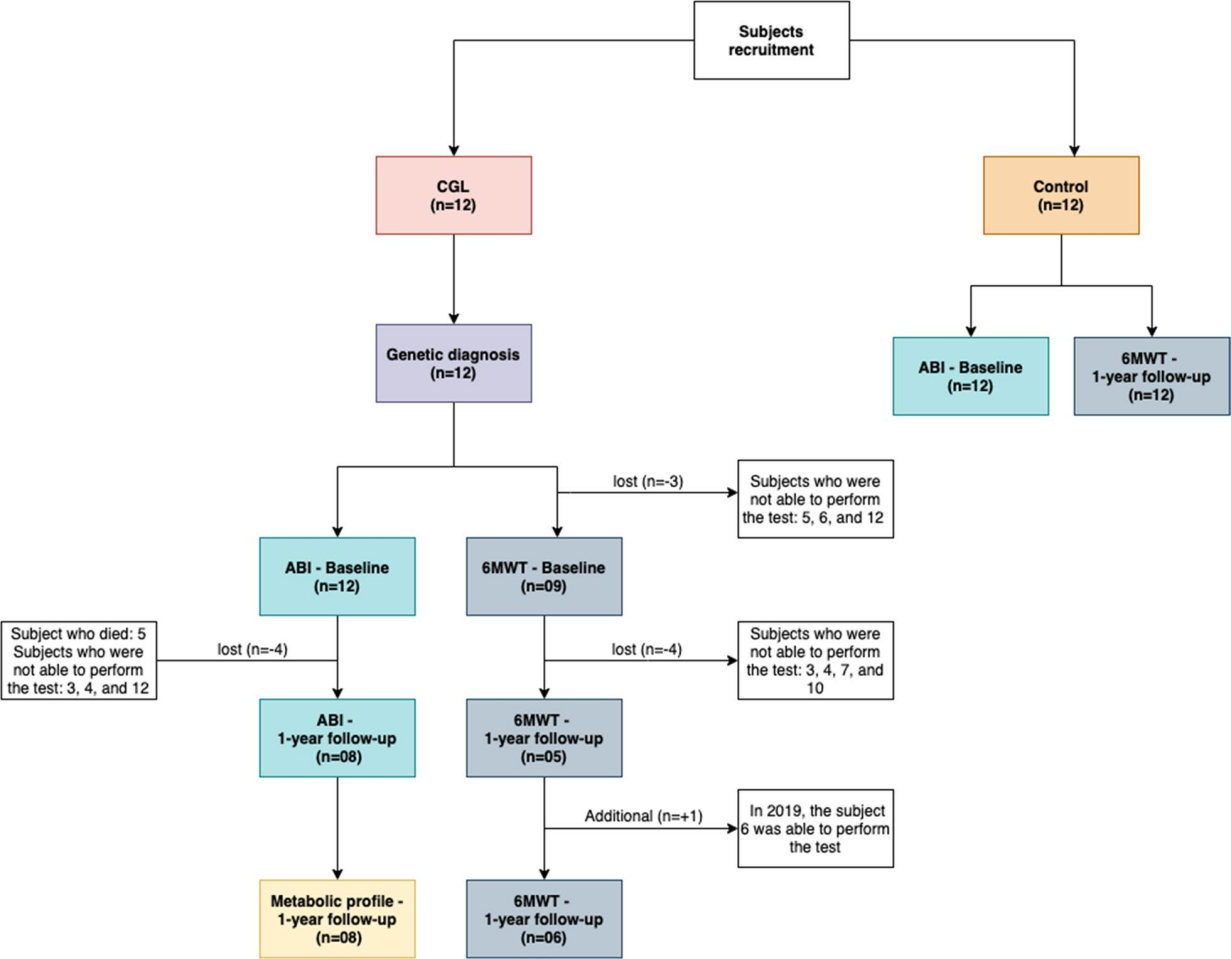
Flowchart of this research. *CGL* Congenital Generalized Lipodystrophy, *ABI* ankle-brachial index, *6MWT* 6-min walk test

The functional exercise capacity and the cardiovascular response (CV) to submaximal exercise were evaluated by the 6-min walk test (6MWT) following the recommendations of the European Respiratory Society/American Thoracic Society (ERS/ATS) [22, 23]. Subjects were instructed to walk as for as they could for 6 min. They performed a single walk test in a plane passage of 30 m in length, in accordance to the ERS/ATS [23]. The same investigator performed measurements by assessing the total distance walked by the subject at the end of the test. The result is expressed as a walked distance (6MWD) in meters and is compared with the predicted values (predict%) for the Brazilian population [24]. For 6WMD measurements, only 9 CGL individuals participated in 2018, and 4 were lost to follow-up in the 2019 data collection (subjects 3, 4, 7, and 10), as indicated in the flowchart (Fig. 1). The 6WMT reference values were calculated according to previous studies of healthy Brazilian subjects: 6MWD (m) = 622.461—(1.846 × age in years) + (61.503 × gender; 0 = female and 1 = male) [24]. We measured systolic blood pressure (SBP), diastolic blood pressure (DBP), heart rate (HR), and pulse oximetry oxygen saturation (SpO_2_) at the beginning and immediately after finishing the 6MWT. The HR and SpO_2_ were measured using a digital finger pulse oximeter (M170 OLED—Shenzhen Fitfaith Technology Co., Ltd). The Borg’s score (0–10) was used to rate perceived exertion and the perceived level of dyspnea [25]. Further, SpO_2_, HR, and Borg’s score were measured every minute for safety and better monitoring of the subjects. At the end of 6WMT, individuals were asked about intermittent claudication, according to Fontaine Classification [26].

### Evaluation of the physical activity level

CGL participants were also asked about their practice and weekly frequency of physical activities, according to the International Physical Activity Questionnaire (IPAQ), by using the 8-question short version [27–29]. This IPAQ version consists of questions about the frequency (days per week) and time (minutes per day) spent on walks and performing activities involving moderate and intense physical effort in 4 domains (work, commuting to work, household duties, and leisure). The main investigator applied the IPAQ questionnaire. The physical activity level was classified as sedentary, irregularly active B, irregularly active A, active, or very active, as previously described [27–29].

### Ankle-brachial index (ABI) measurement

Two experienced technicians assessed the ABI according to the American College of Cardiology Foundation/American Heart Association (ACCF/AHA) Task Force (2016) [30]. We evaluated ABI in 2018 and one year later. Before measurements, subjects remained to lie down for 5 min in the supine position. To assess systolic blood pressure (SBP) of both limbs (in mmHg), we used a portable Doppler device (model DV-2001; MEDPEJ, *São Paulo*, Brazil) that was placed on the brachial arterial in the cubital fossa and placed on the posterior tibial or dorsal foot arteries. The ABI value was obtained by the ratio between the highest SBP value of the lower limbs and the higher value of the brachial artery pressures [30, 31]. The same investigators performed the measurements. Subjects with 1.0 ≤ ABI ≤ 1.4 were considered normal; subjects who had an ABI ≤ 0.90 were categorized as having PAD; subjects with 0.91 ≤ ABI ≤ 0.99 were considered borderline; and ABI > 1.4 were considered non-compressible values, as defined in the 2016 ACCF/AHA [30]. Subjects with ABI above 1.4 were categorized as abnormal PAD. Therefore, healthy subjects with predicted values lower than 1.0 or higher than 1.4 were considered unsuitable and, therefore, were excluded. ABI measurements of 4 CGL individuals were lost to follow-up: 1 had died (subject 5), and three did not participate in the 2019 data collection (subjects 3, 4, and 12).

### Statistical analysis

The Shapiro–Wilk test was used to examine normality data distribution. Continuous and normally distributed variables are expressed as mean ± standard deviation (SD). Proportions are presented as numbers (%) for categorical variables. Associations between categorical variables were analyzed by the chi-square test. For unpaired tests, the comparisons between variables with Gaussian and non-Gaussian distribution were performed by T-test and Mann–Whitney U test, respectively. For paired tests, the comparisons between variables with Gaussian and non-Gaussian distribution were performed by T-test and Wilcoxon matched-pairs test, respectively. Multiple comparisons were performed using ANOVA or Kruskal–Wallis test for Gaussian and non-Gaussian distribution, respectively.

To evaluate the correlation among ABI, 6MWD, and biochemical parameters, Pearson and Spearman correlation coefficients were used, according to the Gaussian or non-Gaussian distribution, respectively. Multiple linear regression was performed to verify the influence of multiple variables on ABI and 6MWD. For all analyses, we used GraphPad Prism software version 8.0, and the statistical significance was set at *p* < 0.05.

## Results

Twelve CGL subjects were studied (8 women—67%). The study design is shown in Fig. 1. Nine CGL subjects were type 2 (75%): 8 presenting a specific homozygous variant in the *BSCL2* gene (c.325dupA), and 1 was a compound heterozygous for the *BSCL2* gene (c.325dupA and the intronic variant c.574-2A > G). Three CGL patients (25%) were type 1, 1 presenting the c.317-588del (case 2) and 2 siblings having the c.646A > T variant in the *AGPAT2* gene (cases 3 and 4). Clinical, metabolic, and genetic data were described (Tables 1, 2).

**Table 1:** Clinical and genetic data of CGL subjects

Case	Gender/age (years)	Mutated gene	Mutation type	Comorbidities	Drugs
1	♂/18	*BSCL2*	*c.325dupA*	HT	None
2	♀/20	*AGPAT2*	*c.317-588del*	DM, HT, SH	ISL, MLP
3*^#^	♀/20	*AGPAT2*	*c.646 C* > *T*	DM, HT, SH	MTF
4*^#^	♀/24	*AGPAT2*	*c.646 C* > *T*	DM, HT, SH, RMW	ISL, MLP
5*^#^	♀/24	*BSCL2*	*c.325dupA*	DM, HT	None
6	♀/27	*BSCL2*	*c.325dupA*	DM, HT	ISL
7*	♂/27	*BSCL2*	*c.325dupA*	DM, HT, RMW	MTF, HCT, SXG, CPF, RMP, MLP
8	♀/31	*BSCL2*	*c.325dupA*	DM, AH, HT, SH, RMW	ISL, MLP
9	♂/32	*BSCL2*	*c.325dupA*	DM, AH, HT, SH, RMW	ISL, SXG, MTF, RMP, MLP
10	♀/33	*BSCL2*	*c.325dupA*	HT	None
11	♂/34	*BSCL2*	*c.325dupA/ c.574-2A* > *G*	RMW	MTF, SVT
12*^#^	♀/46	*BSCL2*	*c.325dupA*	DM, HT, RMW	ISL

*DM* diabetes *mellitus*, *AH* arterial hypertension, *HT* hypertriglyceridemia, *SH* steatohepatitis, *KF* kidney failure, *♀* females, *♂* males

*Subjects who participated in 2018 but were lost to follow-up in 2019 for the 6MWT test

^#^Subjects who participated in 2018 but were lost to follow-up in 2019 for the ABI test. *MTF* Metformin, *MLP* Metreleptin, *HCT* Hydrochlorothiazide, *SXG* Saxagliptin, *CPF* Ciprofibrate, *RMP* Ramipril, *Insulin* ISL, *SVT* Sinvastatin, *RMW* Respiratory Muscle Weakness

**Table 2:** Physiological and metabolic data of CGL subjects

	Baseline (n = 12)	1-year follow-up (n = 8)	Control (n = 12)	*p*^a^	*p*^b^	*p*^c^
Female, n (%)	8 (67)	4 (50)	8 (67)	> 0.999	0.745	0.456
Age (years)	28 ± 7.8	28.5 ± 7.9	27.5 ± 7.6	0.990	0.947	0.981
Height (m)	1.63 ± 0.09	1.63 ± 0.09	1.65 ± 0.09	0.960	0.949	0.998
Weight (kg)	55.7 ± 12.8	56.9 ± 12.2	70.4 ± 2.5	0.022	0.030	0.966
BMI (kg/m^2^)	20.85 ± 0.9	21. ± 2.8	26.4 ± 1.08	0.002	0.001	0.989
DM, n (%)	9 (75)	4 (50)	0 (0)	0.000	0.006	0.250
Fasting glycemia (mg/dL)	155.30 ± 115.8	160.6 ± 108.4	NA	–	–	0.148
Serum triglycerides (mg/dL)	226.3 ± 186.6	141.4 ± 67.1	NA	–	–	0.382
Total cholesterol (mg/dL)	162.9 ± 68	163.1 ± 42.6	NA	–	–	0.250
VLDL-c (mg/dL)	45.2 ± 37.3	25.38 ± 8.1	NA	–	–	0.250
LDL-c (mg/dL)	79.6 ± 34.7	114.9 ± 35.5	NA	–	–	0.015
HDL-c (mg/dL)	32.6 ± 9.5	22.8 ± 6.9	NA	–	–	0.125
Non-HDL-c (mg/dL)	127. 10 ± 73.5	140.3 ± 39.6	NA	–	–	0.031

*p* values were based on independent t-tests. For categorical variables, the *p* value was calculated using the chi-square test. *p*^a^: Comparison between Control and CGL—Baseline. *p*^b^: Comparison between Control and CGL—1-year follow-up. *p*^c^: Comparison between CGL—Baseline and 1-year follow-up. *p* values were based on independent unpaired t-tests or Mann–Whitney test. *BMI* Body Mass Index, *DM* diabetes *mellitus*, *NA* Not available. Baseline peripheral blood of CGL subjects was collected on a different day of ABI and 6WMT data collection. CGL metabolic data from 1-year follow-up were used for correlation analysis since peripheral blood, ABI, and 6WMT were obtained on the same day

The weight and BMI between CGL and control groups were statistically different and 9 (75%) CGL subjects presented diabetes (Table 2). Three CGL individuals (25%) were classified as sedentary, 6 patients (50%) were active, 2 (17%) were irregularly active A, and 1 (8%) was irregularly active B, indicating that the CGL group was mainly active. None of the CGL subjects reported claudication during the 6MWT. The CGL subjects who presented ABI ≤ 0.90 were in the early stages of PAD (Stage I, asymptomatic, according to the Fontaine classification). While no differences were found in fasting glycemia, serum triglycerides, total cholesterol, VLDL-c, and HDL-c levels in CGL subjects, LDL-c and non-HDL-c levels were higher at 1-year follow-up. Since peripheral blood, ABI, and 6WMT were obtained on the same day, CGL metabolic data from 1-year follow-up were used for correlation analysis.

**Fig. 2:**
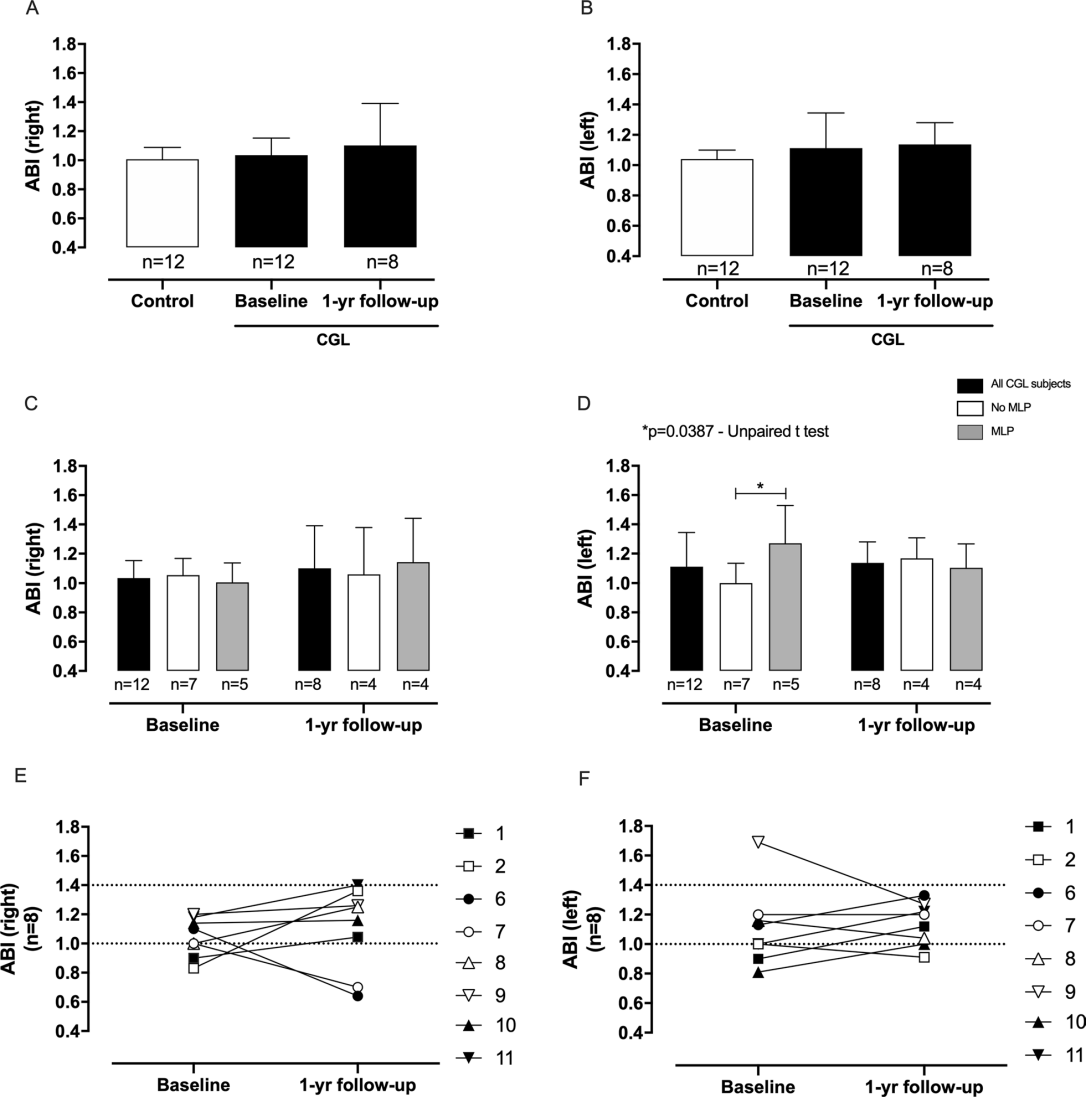
The ankle-brachial index (ABI) values of CGL subjects at baseline and 1-year follow-up. **A** right ABI and **B** left ABI indexes compared to the healthy individuals. **C** right ABI and **D** left ABI indexes according to the use of metreleptin (MLP). ABI indexes are expressed as arbitrary values and the results are represented as the mean ± SD. **E** right ABI and **F** left ABI indexes only of CGL subjects who participated at 1-year follow-up. Filled and empty shapes for each CGL subject indicate without and with MLP replacement, respectively. ABI indexes are expressed as arbitrary values. The differences were considered statistically significant when *p < 0.05 using the unpaired Student’s *t*-test. The significance ranges were represented by: * 0.05 > *p* ≥ 0.01

At baseline and 1-year follow-up, no changes were found in the ABI compared to the controls (Fig. 2A, B and Table 3). We also stratified the CGL subjects according to the use of MLP to better understand the role of MLP in PAD development and/or rescue. Our data suggest that MLP replacement did not change the ABI measures (Fig. 2C–F and Additional file 1: Table S1). We found that only 25% (n = 3) of CGL subjects presented PAD and this finding was not dependent on MLP (Fig. 2E, F).

**Table 3:** ABI and 6MWD measurements (mean ± SD and [95% confidence interval]) in healthy control and all CGL subjects at baseline and 1-year follow-up

	Control	CGL—Baseline	CGL—1-year follow-up	*p*^a^	*p*^b^	*p*^c^
ABI (right)	(n = 12)	(n = 12)	(n = 8)			
	1.06 ± 0.08 [0.95–1.06]	1.03 ± 0.11 [0.96–1.10]	1.10 ± 0.28 [0.86–1.34]	0.509	0.309	0.478
ABI (left)	(n = 12)	(n = 12)	(n = 8)			
	1.04 ± 0.05 [1.00–1.08]	1.11 ± 0.23 [0.96–1.25]	1.13 ± 0.14 [1.01–1.25]	0.324	0.060	0.799
6MWD (meters)	(n = 12)	(n = 9)	(n = 6)			
	515.2 ± 68.48 [471.7–558.7]	435.1 ± 85.78 [369.2–501.0]	537.5 ± 103.3 [429.1–645.9]	0.027	0.588	0.056
6MWD (predict%)	(n = 12)	(n = 9)	(n = 6)			
	87.05 ± 10.93 [80.10–93.99]	72.19 ± 12.43 [62.64–81.75]	88.96 ± 14.48 [73.77–104.2]	0.009	0.757	0.032

*ABI* Ankle-Brachial Index, *6MWD* 6-min walk distance. *p*^a^: Comparison between Control and CGL—Baseline. *p*^b^: Comparison between Control and CGL—1-year follow-up. *p*^c^: Comparison between CGL—Baseline and 1-year follow-up. *p* values were based on independent unpaired t-tests or Mann–Whitney test

**Fig. 3:**
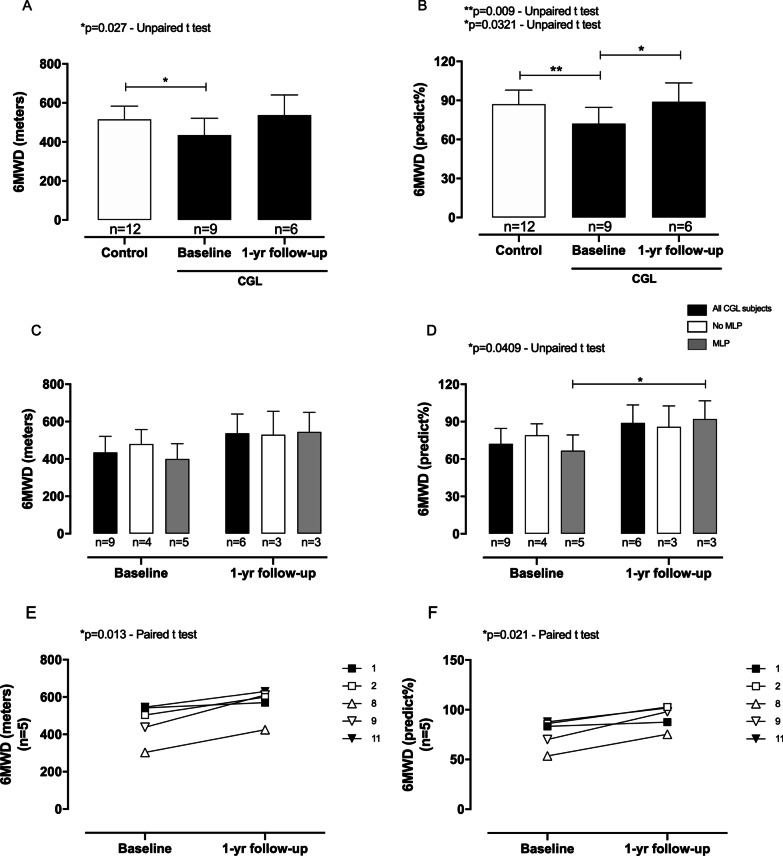
The 6-min walk distance (6MWD) values of CGL subjects at baseline and 1-year follow-up. **A** 6MWD (meters) and **B** 6MWD (predict%) values compared to healthy individuals. **C** 6MWD (meters) and **D** 6MWD (predict%) values according to the use of metreleptin (MLP). The values are represented as the mean ± SD. **E** 6MWD (meters) and **F** 6MWD (predict%) values only of CGL subjects who participated at 1-year follow-up. Filled and empty shapes for each CGL subject indicate without and with MLP replacement, respectively. The differences were considered statistically significant when *p < 0.05 using the unpaired Student’s *t*-test. The significance ranges were represented by: * 0.05 > *p* ≥ 0.01, and ** 0.01 > *p* ≥ 0.001

**Fig. 4:**
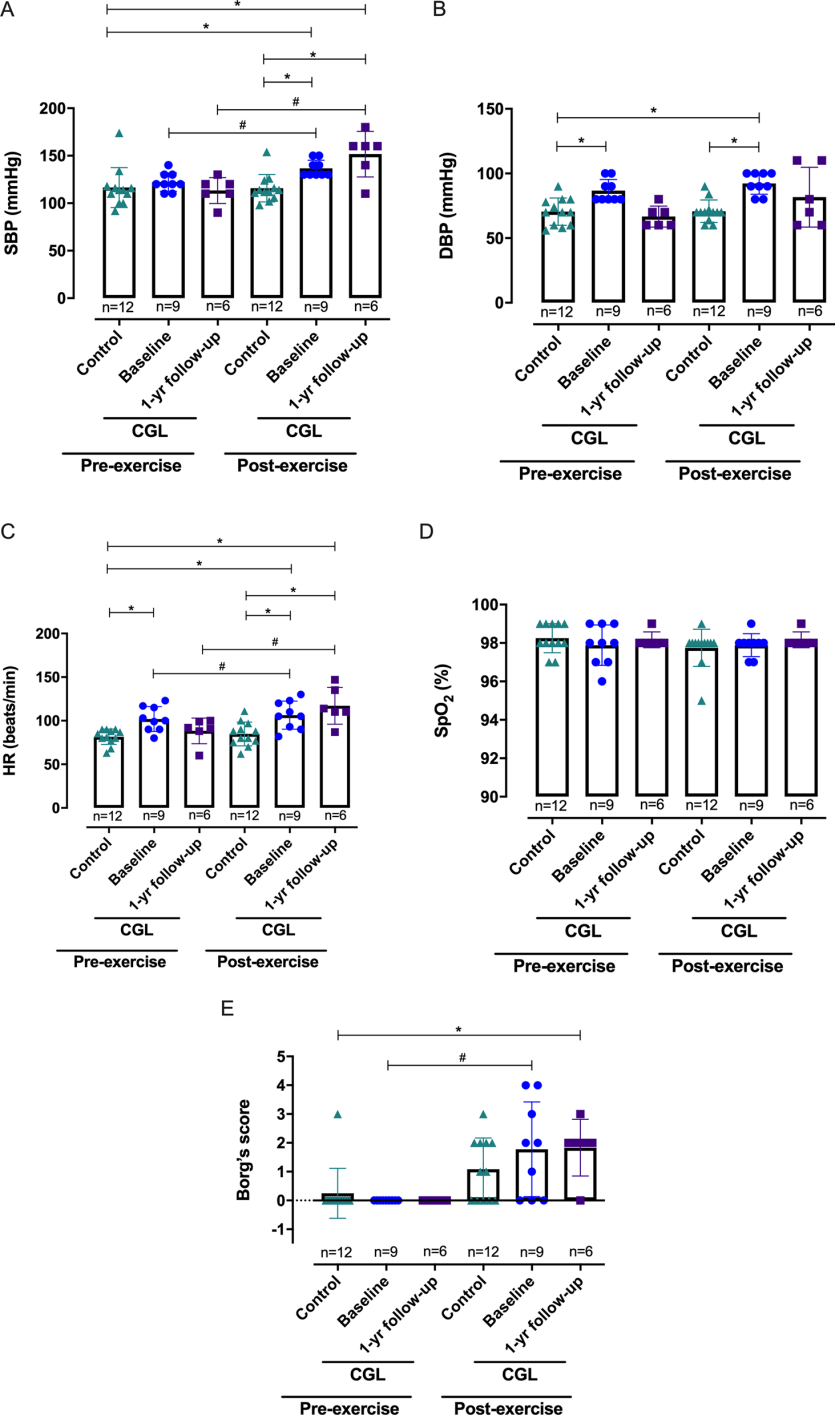
CV variables of CGL and healthy subjects before and after the 6-min walk test (6MWT). **A** Systolic blood pressure (SBP), **B** Diastolic blood pressure (DBP), **C** Heart rate (HR), **D** Oxygen saturation (SpO_2_), and **E** Borg’s score values in the CGL group are from baseline and 1-year follow-up. Multiple comparisons were performed using ANOVA or Kruskal–Wallis test. Intra-group analyses were made using a paired Student’s t-test or Wilcoxon matched-pairs test. The differences were considered statistically significant when *p < 0.05 (for ANOVA or Kruskal–Wallis test) and ^#^*p* < 0.05 (for paired Student’s *t*-test or Wilcoxon matched-pairs test). The significance ranges were represented by: */^#^ 0.05 > *p* ≥ 0.01

The walked distance (6MWD) by the CGL group at baseline was reduced compared with the control group (Fig. 3A, B), and the former presented an increase in the 6MWD at 1-year follow-up (Table 3), which seems to be associated with MLP replacement (Fig. 3C–F). The CV variables revealed that the CGL group presented an increased post-exercise SBP after the 6MWT compared with pre- and post-exercise SBP for the control group (Fig. 4A and Table 4). CGL intra-group analysis showed a higher post-exercise SBP after 6MWD at baseline and 1-year follow-up than the pre-exercise SBP (Fig. 4A and Table 4). Concerning the diastolic pressure, the pre-exercise DBP was higher in the CGL than in the control group (Fig. 4B and Table 4). Further, the post-exercise DBP was higher in the CGL group in the first year compared with the pre- and post-exercise values for controls (Fig. 4B). Pre- and post-exercise HR did not change in the healthy volunteers (Fig. 4C and Table 4), but CGL subjects presented a higher pre-exercise HR than control individuals (Fig. 4C and Table 4). Increased post-exercise HR after 6MWT at the baseline and 1-year follow-up were also seen in the CGL group, compared with the pre-exercise HR for their group (Fig. 4C and Table 4). No differences were found for O_2_ saturation in both groups before and after the 6MWT (Fig. 4D and Table 4). According to Borg’s score, subjective dyspnea scores revealed no differences among healthy and CGL subjects after the 6MWT (Fig. 4E and Table 4). However, at the baseline, CGL intra-group analysis revealed a higher post-exercise Borg’s score than the pre-exercise score. At 1-year follow-up, CGL showed a higher post-exercise Borg’s score than the pre-exercise score of controls (Fig. 4E and Table 4).

**Table 4:** CV variables (mean ± SD and [95% confidence interval]) in CGL subjects and healthy volunteers (Control) before and after 6MWT at baseline and 1-year follow-up

	Control (n = 12)	CGL—Baseline (n = 9)	CGL—1-year follow-up (n = 6)	*p*^a^	*p*^b^	*p*^c^
*SBP*						
Pre-exercise	116.50 ± 21.01 [103.1–129.9]	122.20 ± 9.71 [114.8–129.7]	113.30 ± 13.66 [99.0–127.7]	0.459	0.867	0.162
Post-exercise	115.80 ± 14.44 [106.6–124.9]	136.70 ± 8.66 [130.0–143.3]	151.70 ± 24.01 [126.5–176.9]	0.001	0.012	0.075
*p*^*#*^	0.889	0.003	0.002			
*DBP*						
Pre-exercise	70.50 ± 10.52 [63.82–77.18]	86.67 ± 8.66 [80.01–93.32]	66.67 ± 8.16 [58.10–75.24]	0.001	0.447	0.000
Post-exercise	70.83 ± 8.67 [65.32–76.34]	92.22 ± 8.33 [85.82–98.63]	81.67 ± 23.17 [57.36–106.0]	0.000	0.583	0.337
*p*^*#*^	0.793	0.312	0.150			
*HR*						
Pre-exercise	81.58 ± 8.79 [76.0–87.17]	101.90 ± 14.23 [90.95–112.8]	88.33 ± 14.65 [72.96–103.70]	0.000	0.055	0.170
Post-exercise	84.83 ± 13.67 [76.15–93.52]	106.40 ± 16.12 [94.06–118.8]	117.20 ± 21.12 [95.00–139.30]	0.003	0.001	0.284
*p*^*#*^	0.321	0.003	0.031			
*SpO*_*2*_						
Pre-exercise	98.25 ± 0.75 [97.77–98.73]	97.89 ± 1.05 [97.08–98.70]	98.17 ± 0.40 [97.74–98.60]	0.370	0.795	0.719
Post-exercise	97.75 ± 0.96 [97.14–98.36]	97.89 ± 0.6 [97.43–98.35]	98.17 ± 0.40 [97.14–98.60]	0.709	0.490	0.633
*p*^*#*^	0.234	> 0.999	> 0.999			
*Borg’s score*						
Pre-exercise	0.25 ± 0.86 [-0.30–0.39]	0 [0–0]	0 [0–0]	0.400	> 0.999	> 0.999
Post-exercise	1.08 ± 1.08 [0.80–1.77]	1.77 ± 1.64 [0.51–3.04]	1.83 ± 0.98 [0.80–2.86]	0.256	0.230	0.959
*p*^*#*^	0.062	0.031	0.062			

*SBP* Systolic Blood Pressure (mmHg), *DBP* Diastolic Blood Pressure (mmHg), Heart rate (beats/min), *SpO*_*2*_ Pulse oximetry oxygen saturation, *ABI* Ankle-Brachial Index. 6MWT: 6-min walk test. *p*^a^: Comparison between Control and CGL—Baseline. *p*^b^: Comparison between Control and CGL—1-year follow-up. *p*^c^: Comparison between CGL—Baseline and 1-year follow-up. *p*^a,b,c^ values were based on independent unpaired t-test or Mann–Whitney test. *p*^*#*^ values were based on independent paired t-tests or Wilcoxon matched-pairs test

**Fig. 5:**
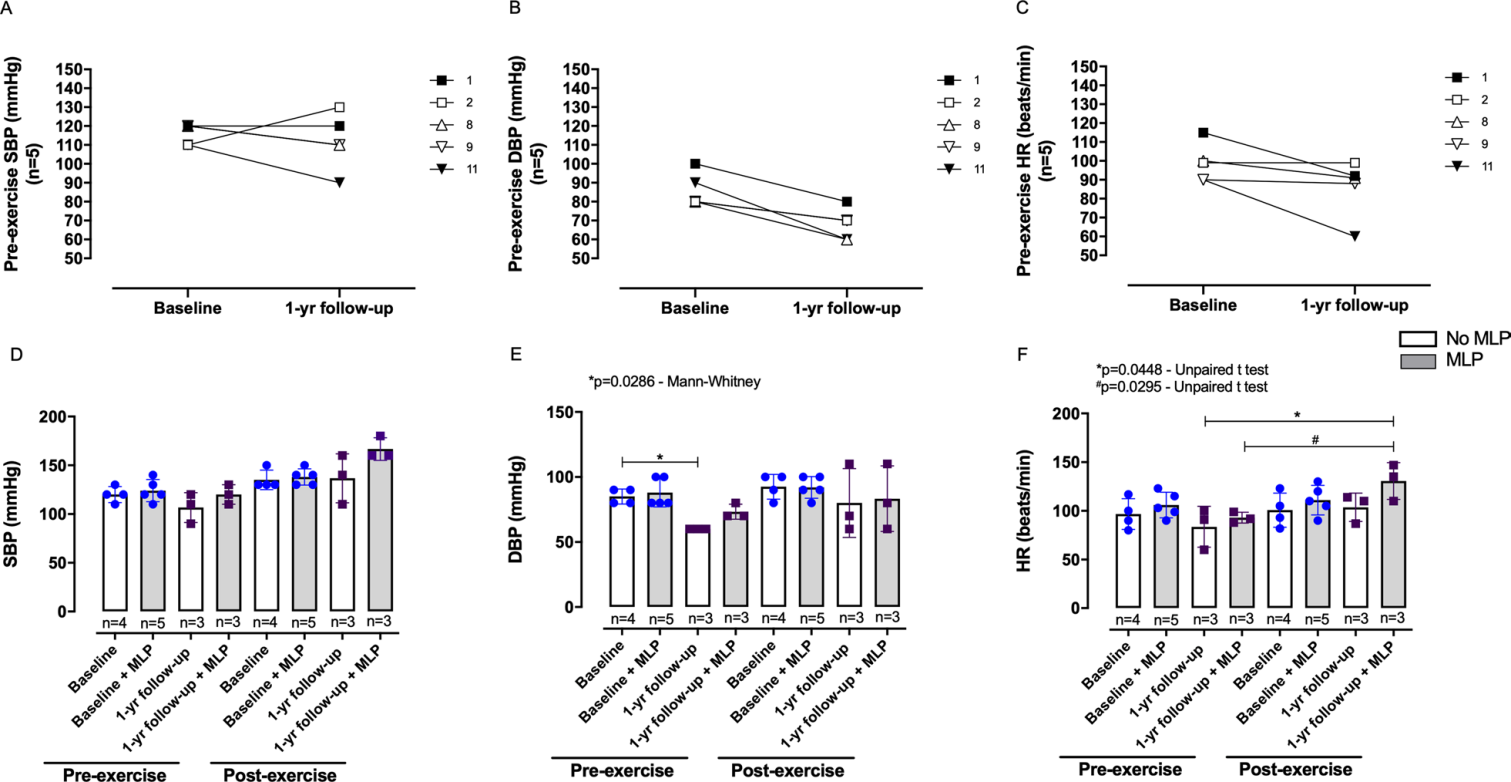
The CV variables of CGL subjects before and after the 6-min walk test (6MWT) according to the use of metreleptin (MLP). **A** Pre-exercise systolic blood pressure (SBP), **B** pre-exercise diastolic blood pressure (DBP), and **C** pre-exercise heart rate (HR) only of CGL subjects who participated at 1-year follow-up. Filled and empty shapes indicate without and with metreleptin (MLP) replacement, respectively. **D** Systolic blood pressure (SBP), **E** Diastolic blood pressure (DBP), and **F** Heart rate (HR) before and after the 6-min walk test (6MWT). The column corresponding to MLP replacement was kept in gray. The values are from 2018 to 2019. The differences were considered statistically significant when *p < 0.05 using unpaired Student’s *t*-test or Mann–Whitney test. The significance ranges were represented by: */# 0.05 > *p* ≥ 0.01

We also investigated the MLP effects on blood pressure only for CGL subjects who participated at 1-year follow-up. The results showed that the pre-exercise DBP but not the SBP decreased (Fig. 5A, B), and no significant differences were observed in the pre-exercise HR (Fig. 5C).

To verify if the reduction observed in DBP was dependent on MLP replacement, we stratified the CGL subjects according to the use of MLP. The results showed that the pre-exercise SBP and DBP did not decrease for CGL subjects who received MLP compared with subjects without MLP replacement (Fig. 5D, E), ratifying the global results shown in Fig. 5A, B. The DBP was significantly decreased at 1-year follow-up in CGL subjects who did not receive MLP replacement (Fig. 5E), indicating that other variables may regulate the DBP, including metabolic parameters and the practice of physical activity. On the other hand, MLP seems to affect CV response since we observed a greater post-exercise HR at 1-year follow-up in CGL subjects who received MLP replacement than the pre-exercise HR of CGL subjects without and with MLP replacement (Fig. 5F).

The variables that maintained significant correlation were 6MWD and right ABI. We found that 6MWD was significantly higher in CGL subjects who presented greater right ABI. Further, metabolic parameters such as glucose, triglycerides, and VLDL-c inversely correlated with right ABI (Additional file 1: Figs. S1 and S2). Additional correlations are shown in the Additional file 1: Fig. S1. The relationship between metabolic parameters and right ABI revealed that glucose and triglycerides explained 95% of the variability in the right ABI (*R*^2^_adjusted_ = 0.9497) (Additional file 1: Table S2).

## Discussion

Cardiovascular disorders, such as hypertension, atherosclerosis, hypertrophic cardiomyopathy, left ventricular hypertrophy, cardiovascular autonomic neuropathy, abnormal autonomic modulation, electrocardiogram disturbances, and cardiac arrhythmias, were previously described for CGL individuals [4, 6, 10–12, 32]. Regarding atherosclerotic vascular complications, Misra et al. (2004) confirmed that only two patients with acquired congenital lipodystrophy (AGL) had PAD [32]. However, due to the rarity of lipodystrophic patients, the etiology of CGL vascular abnormalities is scarce, and data concerning the physical activity performance and its association with CV response and PAD occurrence in CGL remain unknown.

This is the first study that examined the association between functional exercise capacity, CV response, and PAD in individuals with this ultra-rare disease. In our cohort, CGL individuals showed a reduced walked distance (6MWD), and the CV response was greater than controls, indicating an exacerbated exercise effort and impaired exercise performance in the CGL group. Most CGL subjects presented ABI values similar to healthy individuals (1.0 ≤ ABI ≤ 1.4). Conversely, some CGL individuals had right and/or left ABI values ≤ 0.90 (PAD) as well as 0.91 ≤ ABI ≤ 0.99 (borderline PAD). Interestingly, CGL subjects with lower ABI also showed lesser 6MWD, suggesting an association between physical activity performance and cardiovascular risk by the ABI.

The 6MWT has been used to assess functional exercise capacity in chronic respiratory diseases and metabolic disorders, including DM [33–36], since this test is simple and economical and quantifies the ability to perform daily life activities [33, 37]. In people with type 2 DM, although the 6MWT performance was similar compared to healthy controls, they showed greater cardiovascular effort (SBP, DBP, and HR), revealing an impaired CV response [36]. An exacerbated CV response and impaired exercise performance were found in additional studies evaluating type 2 DM individuals [38–40].

Several traditional cardiovascular risk factors, such as hypertension and dyslipidemia, mainly hypertriglyceridemia, are related to a high risk of developing DM and are also found in CGL patients [41–43]. Both comorbidities predispose patients with lipodystrophy to DM, atherosclerosis, and PAD [43, 44]. Despite the high cardiovascular risk in CGL subjects, their main causes of death were liver disease and infection. Cardiovascular complications were the fourth cause of death in our population [1]. The probable reason for this is that cardiovascular disease is chronic, and death in these patients is usually precocious, often before the age of 30 years.

PAD limits exercise capacity even in the absence of intermittent claudication, and exercise training has been shown to improve both walking ability and ABI even in PAD patients with no claudication [45]. We found that none of the CGL subjects presented claudication, even those with PAD and borderline PAD. Further, all CGL patients had an increase in 6MWD at 1-year follow-up. This increase seems to be dependent on MLP since only CGL subjects who received MLP replacement showed a significantly greater 6MWD in an intra-group analysis comparing the baseline and 12 months after MLP replacement. However, more studies are required to elucidate the role of MLP on functional exercise capacity.

Previous investigations concerning the MLP effects on blood pressure in CGL subjects revealed contrasting results. Ebihara et al. (2007) showed no changes in SBP and DBP in 7 CGL subjects throughout the MLP replacement up to 36 months [46]. On the other hand, Brown et al. (2015) revealed a significant decrease in SBP and DBP 12 months after MLP replacement, reinforcing that in CGL subjects MLP does not mediate hypertension as in obese patients [47]. Here, we found no changes in pre-exercise SBP and DBP. When we stratified the CGL group to verify the MLP effect 12 months after replacement, we found that CGL subjects who participated at 1-year follow-up presented a significant reduction in pre-exercise DBP. However, no differences were found when we analyzed CGL individuals who received and did not receive MLP replacement. Additional investigations are needed to understand better the role of MLP in the blood pressure of CGL subjects.

Lipoprotein disturbances have also been associated with increased CV risk [48, 49] and ABI is a marker of systemic atherosclerotic vascular disease [50]. In fact, the association between low ABI and increased cardiovascular mortality risk is well established, indicating that the ABI measurement is an important tool for diagnosing PAD and for monitoring vascular commitments, as well as for predicting myocardial infarction [51–53]. We found a negative correlation between ABI and glucose, triglycerides, and VLDL-c. CGL subjects who presented the higher ABI had lower triglyceride and VLDL-c levels and showed a greater 6MWD. Both LDL-c and non-HDL-c levels were higher at 1-year follow-up in CGL subjects, and ABI was inversely associated with fasting glycemia, serum triglycerides, and VLDL-c levels. It has been postulated that non-HDL-c may be a more potent predictor of mortality associated with CV disease than LDL-c levels [54, 55]. Also, an association was shown between VLDL-c and the thickness of carotid intima-media layers [56]. Further, in peritoneal dialysis patients, who have an atherogenic lipid profile, ABI was inversely associated with VLDL-c levels [45]. Our results agree with these data, suggesting that CGL subjects present lipid physiology related to atherogenic effects in peripheral arteries.

The present study has some limitations. Duplex ultrasound (DUS), computerized tomography angiography (CTA), magnetic resonance angiography (MRA), and catheter angiography may present higher accuracy in diagnosing PAD than the ABI [57]. However, the ABI is an easy, non-invasive clinical test widely used by healthcare professionals in primary and secondary care settings. Moreover, the sample size is a relevant limitation. However, considering that CGL is an ultra-rare disease, our study presented relevant information to improve the knowledge of the clinical aspects of the disease.

## Conclusions

In conclusion, our data reveal impaired functional exercise capacity and greater cardiovascular effort to exercise for people with CGL than healthy individuals, suggesting that 6MWT can be a useful marker of exercise performance in CGL. Data from functional exercise capacity associated with ABI showed that CGL subjects with better exercise performance showed higher ABI, reinforcing that an exercise program in CGL is important to reduce limb symptoms and improve performance and CV response to exercise. In addition, CGL individuals who underwent MLP replacement seem to have increased functional exercise capacity. However, this relationship needs to be further studied in a larger cohort.

## Supplementary Information

**Additional file 1: Table S1.** ABI and 6MWD measurements (mean ± SD and [95% confidence interval]) in all CGL subjects without and with metreleptin (MLP) replacement at baseline and 1 year follow up. **Table S2.** Multiple linear regression analysis with right ABI at 1-year follow-up as the dependent variable in CGL subjects. **Fig. S1.** Correlation among the ankle brachial index (ABI) index, six min walk distance (6MWD) hemodynamic indexes, metabolic, and anthropometric data in CGL subjects at 1 year follow up. (A) The upper panel shows Spearman correlation coefficient values. (B) The lower panel shows the Pearson correlation coefficient values. Variables with non Gaussian distribution were: glucose, 6MWD (predict%), pre exercise HR, pre exercise oxygen saturation (SpO_2_) and post exercise oxygen saturation (SpO_2_) TC: Total cholesterol. TG: Triglycerides. **Fig. S2.** Correlations among the r ight ankle brachial index (ABI) six min walk distance (6MWD) and metabolic parameters in CGL subjects at 1 year follow up. (A) Right ABI positively correlated with 6MWD. (B) Right ABI negatively correlated with glucose. (C) Right ABI negatively correlate d with triglycerides. (D) Right ABI negatively correlated with VLDL c. ABI, 6MWD, glucose, triglycerides, and VLDL c at 1 year follow up were used. r values of a Pearson or Spearman correlation coefficient and p values are included.

## Data Availability

All data generated or analyzed during this study are included in this published article.
