# Supplementary material for: Impaired functional exercise capacity and greater cardiovascular response to the 6-min walk test in congenital generalized lipodystrophy
Source: BMC Cardiovasc Disord. 2022 Aug 25;22:384. doi: 10.1186/s12872-022-02828-x (PMC9414389; doi:10.1186/s12872-022-02828-x)
Supplement: Supplementary file 1 — Additional file 1: Table S1. ABI and 6MWD measurements (mean ± SD and [95% confidence interval]) in all CGL subjects without and with metreleptin (MLP) replacement at baseline and 1 year follow up. Table S2. Multiple linear regression analysis with right ABI at 1-year follow-up as the dependent variable in CGL subjects. Fig. S1. Correlation among the ankle brachial index (ABI) index, six min walk distance (6MWD) hemodynamic indexes, metabolic, and anthropometric data in CGL subjects at 1 year follow up. (A) The upper panel shows Spearman correlation coefficient values. (B) The lower panel shows the Pearson correlation coefficient values. Variables with non Gaussian distribution were: glucose, 6MWD (predict%), pre exercise HR, pre exercise oxygen saturation (SpO2) and post exercise oxygen saturation (SpO2) TC: Total cholesterol. TG: Triglycerides. Fig. S2. Correlations among the r ight ankle brachial index (ABI) six min walk distance (6MWD) and metabolic parameters in CGL subjects at 1 year follow up. (A) Right ABI positively correlated with 6MWD. (B) Right ABI negatively correlated with glucose. (C) Right ABI negatively correlate d with triglycerides. (D) Right ABI negatively correlated with VLDL c. ABI, 6MWD, glucose, triglycerides, and VLDL c at 1 year follow up were used. r values of a Pearson or Spearman correlation coefficient and p values are included. [file 12872_2022_2828_MOESM1_ESM.pdf]

Impaired Functional Exercise Capacity and Greater Cardiovascular Response to the 6-Minute Walk Test in Congenital Generalized Lipodystrophy

Jorge Luiz Dantas de Medeiros[1]¶, Bruno Carneiro Bezerra[2]¶, Helen Rainara Araújo Cruz[2], Katarina Azevedo de Medeiros[2], Maria Eduarda Cardoso de Melo[3], Aquiles Sales Craveiro Sarmento[3], Marcela Abbott Galvão Ururahy[4], Lucymara Fassarella Agnez Lima[3], Alcebíades José dos Santos Neto[5], Josivan Gomes Lima[5], Vanessa Resqueti[1,6], Lucien Peroni Gualdi[2], Guilherme Fregonezi[1,6], Julliane Tamara Araújo de Melo Campos[3*]

Supporting Tables

S1 Table. ABI and 6MWD measurements (mean ± SD and [95% confidence interval]) in all CGL subjects without and with metreleptin (MLP) replacement at baseline and 1-year follow-up.

CGL subjects		Baseline (n/%)	1-year follow-up (n/%)	p^b
**ABI (right)**		**(n = 12)**	**(n = 8)**	
	**No MLP**	**7 (58)**	**4 (50)**	-
		1.05 ± 0.11	1.06 ± 0.31	0.968
		[0.95 - 1.15]	[0.55 - 1.56]	-
	**MLP**	**5 (42)**	**4 (50)**	-
		1.00 ± 0.13	1.14 ± 0.29	0.384
		[0.84 - 1.16]	[0.66 - 1.61]	-
	p^a	0.495	0.721	-
**ABI (left)**		**(n = 12)**	**(n = 8)**	
	**No MLP**	**7 (58)**	**4 (50)**	-
		1.00 ± 0.13	1.16 ± 0.14	0.082
		[0.87 - 1.12]	[0.94 - 1.39]	-
	**MLP**	**5 (42)**	**4 (50)**	-
		1.27 ± 0.25	1.10 ± 0.16	0.305
		[0.94 - 1.59]	[0.84 - 1.36]	-
	p^a	0.038	0.581	-
**6MWD (predict%)**		**(n = 9)**	**(n = 6)**	
	**No MLP**	**4 (44.4)**	**3 (50)**	-
		79.08 ± 9.15	85.88 ± 16.76	0.516
		[62.52 - 93.64]	[44.24 - 127.5]	-
	**MLP**	**5 (55.6)**	**3 (50)**	-
		66.68 ± 12.69	92.04 ± 14.65	0.040
		[50.92 - 82.44]	[55.64 - 128.4]	-
	p^a	0.146	0.656	-

ABI: Ankle-Brachial Index. 6MWD: Six-minute walk distance. p^a and p^b values were based on independent unpaired t-tests. MLP: Metreleptin.

S2 Table. Multiple linear regression analysis with right ABI at 1-year follow-up as the dependent variable in CGL subjects.

Variable	Coefficient β	95% CI	*p*
**Intercept (β0)**	2.373	1.485 to 3.260	0.003
**Glucose (β1)**	-0.001546	-0.002334 to -0.0007573	0.008
**Triglycerides (β2)**	-0.002625	-0.004265 to -0.0009864	0.014
**Age (years) (β3)**	-0.005731	-0.02201 to 0.01055	0.344
**BMI (kg/m^2) (β4)**	-0.02247	-0.06049 to 0.01555	0.156

Adjusted R^2: 0.9497. 95% IC: 95% confidence interval. ABI: Ankle-Brachial Index. BMI: Body Mass Index.

Supporting Images

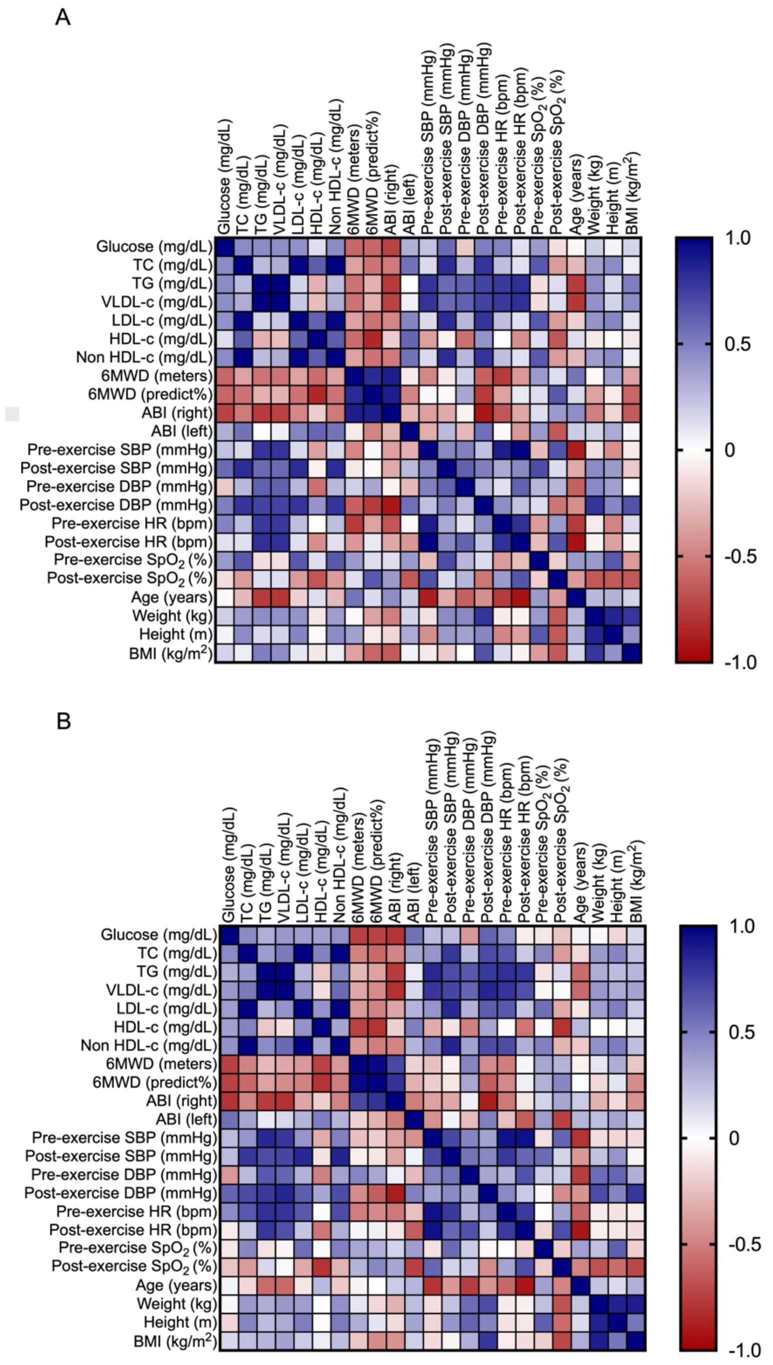

S1 Fig. Correlation among the ankle-brachial index (ABI) index, six-min walk distance (6MWD), hemodynamic indexes, metabolic, and anthropometric data in CGL subjects at 1-year follow-up. (A) The upper panel shows Spearman correlation coefficient values. (B) The lower panel shows the Pearson correlation coefficient values. Variables with non-Gaussian distribution were: glucose, 6MWD (predict%), pre-exercise HR, pre-exercise oxygen saturation (SpO_2), and post-exercise oxygen saturation (SpO_2). TC: Total cholesterol. TG: Triglycerides.

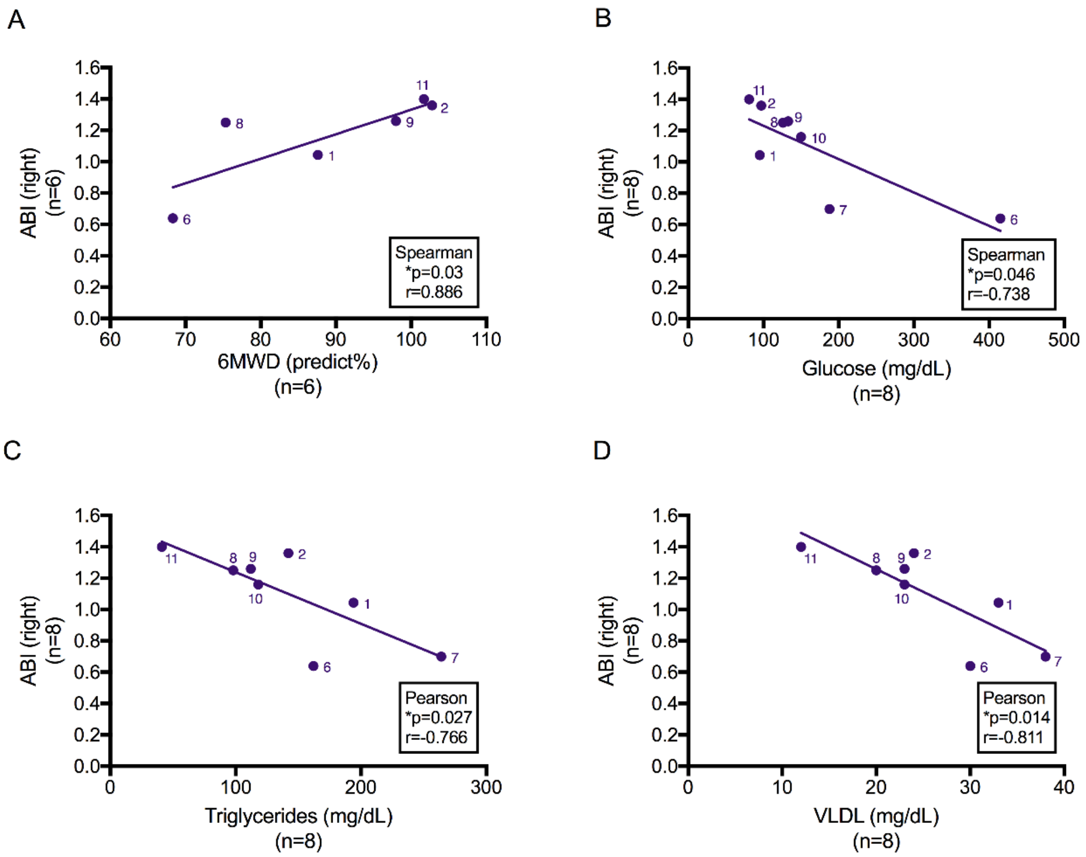

S2 Fig. Correlations among the right ankle-brachial index (ABI), six-min walk distance (6MWD), and metabolic parameters in CGL subjects at 1-year follow-up. (A) Right ABI positively correlated with 6MWD. (B) Right ABI negatively correlated with glucose. (C) Right ABI negatively correlated with triglycerides. (D) Right ABI negatively correlated with VLDL-c. ABI, 6MWD, glucose, triglycerides, and VLDL-c at 1-year follow-up were used. *r* values of a Pearson or Spearman correlation coefficient and *p* values are included.
